# Matrix-R Theory: A Simple Generic Method to Improve RGB-Guided Spectral Recovery Algorithms †

**DOI:** 10.3390/s25247662

**Published:** 2025-12-17

**Authors:** Graham D. Finlayson, Yi-Tun Lin, Abdullah Kucuk

**Affiliations:** School of Computing Science, University of East Anglia, Norwich NR4 7TJ, UK

**Keywords:** spectral reconstruction, spectral super-resolution, pan-sharpening, spectral image fusion, Matrix-R

## Abstract

RGB-guided spectral recovery algorithms include both spectral reconstruction (SR) methods that map image RGBs to spectra and pan-sharpening (PS) methods, where an RGB image is used to guide the upsampling of a low-resolution spectral image. In this paper, we exploit Matrix-R theory in developing a post-processing algorithm that, when applied to the outputs of any and all spectral recovery algorithms, almost always improves their spectral recovery accuracy (and never makes it worse). In Matrix-R theory, any spectrum can be decomposed into a component—called the fundamental metamer—in the space spanned by the spectral sensitivities and a second component—the metameric black—that is orthogonal to this subspace. In our post-processing algorithm, we substitute the correct fundamental metamer, which we calculate directly from the RGB image, for the estimated (and generally incorrect) fundamental metamer that is returned by a spectral recovery algorithm. Significantly, we prove that substituting the correct fundamental metamer always reduces the recovery error. Further, if the spectra in a target application are known to be well described by a linear model of low dimension, then our Matrix-R post-processing algorithm can also exploit this additional physical constraint. In experiments, we demonstrate that our Matrix-R post-processing improves the performance of a variety of spectral reconstruction and pan-sharpening algorithms.

## 1. Introduction

Compared to RGB cameras where there are only three values per pixel [1], hyperspectral and multispectral cameras record more detailed spectral signatures from a scene. The additional information in a multi- or hyperspectral capture has been shown to be important in applications ranging from medical imaging [2,3], remote sensing [4,5], food processing [6,7,8] and art conservation [9,10]. However, the higher price tag, lower spatial resolution, longer integration time and/or bulkiness of spectral imagers limits their practical use.

There are many algorithms exploiting statistical regression and machine learning that attempt to recover high-quality spectral images from (or with the help of) the RGB images. In spectral reconstruction (SR), hyperspectral images are recovered directly from their RGB image counterparts. Here, a ground-truth dataset of paired hyperspectral and RGB data is used to train the SR method. Example approaches include regression (pixel-based one-to-one mapping) [11,12,13,14] and deep learning-based algorithms (patch-by-patch mapping) [15,16,17].

In RGB pan-sharpening (PS), a low-resolution hyperspectral or multispectral image is upsampled to full resolution using a full-resolution RGB image as a guide. The term “sharpened” comes from the fact that if we naively upsampled the images (e.g., using bilinear upsampling), the spectral image would appear blurred relative to the RGB counterpart. When pan-sharpening works well, it looks like the low-resolution spectral image has been sharpened.

There are two variants of RGB-guided pan-sharpening. When we upsample a low-resolution hyperspectral image (where finely sampled spectra are measured at every pixel), we call it *hyperspectral* pan-sharpening. Often, however, the image we wish to upsample is still a multichannel image but with more channels than a 3-channel RGB image. In this case, we call this *multispectral* pan-sharpening. While the image data is different, the algorithms themselves can often be applied to both the hyper- and multispectral capture scenarios, e.g., [18,19,20,21]. Together, SR and PS are examples of RGB-guided spectral recovery algorithms.

Unlike most recent works in spectral recovery, in this paper, we take a step back and ask a fundamental question: “Given a recorded RGB response and assuming the camera sensitivities are known, are there fundamental properties that any recovered spectrum must adhere to?” In 1953, Wyszescki [22] first described that each radiance spectrum is composed of a fundamental component intrinsic to its RGB tristimulus response (later called the “fundamental metamer”) and its “metameric black”. The fundamental metamer integrates to the same given RGB and the black component integrates to zero RGB, [0, 0, 0] (which is where “black” comes from).

Given an RGB of a spectrum and the device spectral sensitivities, we can find the *actual* fundamental metamer defined to be the spectrum in the span of the spectral sensitivities of the camera sensors that projects to the given RGB. Then, we call the projection of a given estimated spectrum (estimated by an SR or PS algorithm) onto the same 3-dimensional spectral subspace spanned by the spectral sensitivities the *estimated* fundamental metamer. We are being careful in our definitions here as, generally, a spectral recovery algorithm—even though the actual RGB is known—will recover a spectrum where the estimated fundamental metamer is not equal to the actual fundamental metamer. One consequence of this result is that when the estimated spectrum, from a given input RGB, is numerically integrated with the camera sensitivities, the calculated output RGB will not be the same as the input [23,24]. This also means that the estimated spectrum, for most prior-art algorithms, must be the wrong answer.

**Figure 1:**
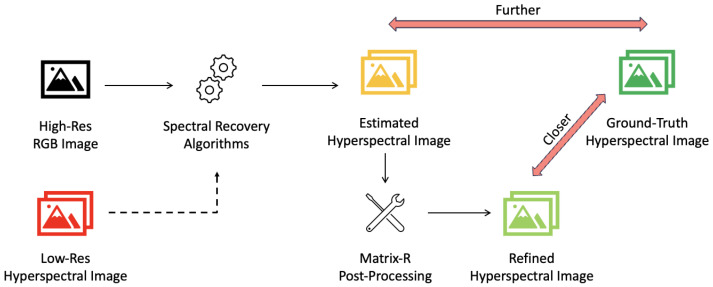
We demonstrate how the Matrix-R post-processing method works. First, either RGB images alone (in the spectral reconstruction case) or RGB images combined with low-resolution hyperspectral images (in pan-sharpening) are fed into existing spectral recovery algorithms. The output images are then enhanced by the Matrix-R post-processing algorithm. These refined images consistently achieve greater accuracy and are closer to the ground-truth compared to the initial estimates.

As we further apply Matrix-R theory to the application of spectral recovery, we learn that a given RGB suggests its corresponding spectrum must have a unique fundamental metamer but can have different metameric blacks. This said, we would argue that the problem of spectral recovery should be about recovering the metameric black because for a given RGB, the fundamental metamer is uniquely prescribed by the, assumed known, spectral sensitivities of the camera. Yet, curiously, the vast majority of algorithms, e.g., [15,21,24,25,26,27], formulate spectral recovery as minimizing a figure of merit (e.g., RMSE) for a given dataset. And, in so doing, the individual recovered spectra can have the wrong estimated fundamental metamers. In a couple of recent works the idea that spectral reconstruction should focus on recovering the metameric black has been investigated with promising results [23,28,29]. In this paper, we show how, instead of re-architecting and retraining already deployed algorithms, we can always improve them via a simple post-processing step, as presented in Figure 1. Here, the output of the existing spectral recovery algorithms is refined by the Matrix-R post-processing step, bringing it closer to the ground-truth hyperspectral images. Low-resolution hyperspectral images are indicated with a dashed arrow, as they are only used by the pan-sharpening algorithms; otherwise, if high-resolution RGB images alone are used, it is referred to as spectral reconstruction.

**Figure 2:**
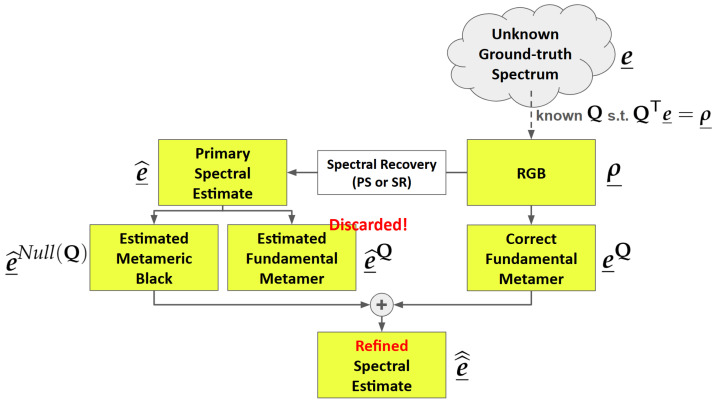
A spectrum of light measured by a camera system Q results in an RGB. In SR, an estimated spectrum is returned directly from analyzing the RGB image. In PS, low-res hyperspectral image can also guide spectral estimation. We group PS and SR algorithms in the single “spectral recovery algorithm” box. The estimated spectrum is decomposed into estimated metameric black and fundamental metamer components. Combining the correct fundamental metamer, calculated directly from the RGB [31], with this estimated metameric black returns a refined estimate of the spectrum. Refining a spectral estimate in this way is called “Matrix-R post-processing”. See the text for a description of the mathematical notation, while this figure serves as a glossary of important notations in this paper.

In more technical terms, Figure 2 illustrates how our post-processing method is deployed. First, we denote the targeted (but unknown) ground-truth spectrum as $\underline{e}$, which forms an RGB $\underline{\rho }$ through the camera system Q. Here, $\underline{e}$ is an *n*-dimensional vector corresponding to the measurements made across a range of *n* sample wavelengths. A spectral reconstruction or pan-sharpening algorithm returns a primary estimation of the spectrum, denoted as $\hat{\underline{e}}$. In our method, $\hat{\underline{e}}$ is uniquely decomposed into estimated fundamental metamer and metameric black components, respectively denoted ${\underline{\hat{e}}}^{Q}$ and ${\underline{\hat{e}}}^{\mathrm{Null}(Q)}$. The notation ^**Q**^ indicates that the decomposition is done with respect to the camera system Q, and the metameric black component lies in the null space [30] of Q.

Then, though the ground-truth $\underline{e}$ is unknown, given the observed RGB $\underline{\rho }$ and the camera system Q, we can still derive its fundamental metamer, ${\underline{e}}^{Q}$ [31]. Finally, the refined spectral estimate is calculated as 1pt6.5pte_^^=e_Q+e^_Null(Q). A key result of this paper is to prove that the refined estimate must be at least as close to the actual ground-truth than the primary estimate made by the SR or PS algorithms and it is, empirically, often much closer. This post-processing is generic and can be applied to all algorithms, using classical or deep learning approaches, reported in the literature.

We call our method “Matrix-R post-processing” because the algorithm illustrated in Figure 2 depends on a particular projector matrix R [22,31] and the post-processing can be described in terms of simple matrix multiplications in terms of R. The operation of matrix R is summarized in the next section, and our post-processing algorithm and a proof of its efficacy are presented in Section 3.

We go on to develop the underlying theory when additional constraints are known about the spectra in a scene. Specifically, it is well known that spectral reflectances are smooth and are well described by low-dimensional linear models (of around 6 to 8 [32,33]). Concomitantly, the spectra, in a scene illuminated by a single dominant light, will have the same dimension as the reflectances (though they will span a different subspace as light spectra are often not smooth). We show how we extend our method to incorporate this linear basis constraint into the Matrix-R theory.

We empirically test our post-processing algorithm on several spectral reconstruction and spectral pan-sharpening algorithms. In all cases we improve the recovery error. When the spectra in a scene belong to a low-dimensional linear basis, our post-processing algorithm delivers an even larger reduction in the recovery error.

We also consider the case where we attempt not to recover full spectra but rather a multispectral representation (i.e., the multispectral pan-sharpening), where given an RGB guide and an *m* channel low-dimensional measurement of the scene (where m≫3), we seek to recover the full-res *m*-channel multispectral image. We show how our developed theory can also be applied in this case and include a final small experimental section as a proof of concept.

This paper makes three key contributions:

- While most studies in the field of spectral recovery are dedicated to presenting new models to solve the problem, the methods in this paper are designed to improve all existing and future methods.
- Our approach is fundamental in nature—the guaranteed improvement of performance by our methods is grounded in mathematical proofs rather than empirical research.
- The methods are developed as a post-processing process, which means no change or retraining needs to be done to apply to a given algorithm, including the off-the-shelves black-box solutions. We also note that our proposed methods are *simple*; that is, it does not incur excessive processing.

## 2. Background

### 2.1. Color and Spectral Image Formation

A radiance spectrum reflected/emitted from a scene is written as the continuous spectral function E(λ). Using a hyperspectral imaging device, we can measure E(λ) at finely sampled wavelengths. Assuming we sample *n* points within the 400 to 700 nm visible range (n≫3), we get an *n*-dimensional vector of measurements $\underline{e}={\left[E\left(\lambda_{1}\right),E\left(\lambda_{2}\right),\cdots ,E\left(\lambda_{n}\right)\right]}^{T}$. Often n=31, where 10 nm sampling is used [15,24,25]. Here and throughout this paper, ^**T**^ denotes the transpose operator.

In contrast, either an RGB or a multispectral camera uses multiple colored sensors with different spectral sensitivities. Let us denote the *k*th-channel spectral sensitivity as $Q_{k}\left(\lambda \right)$ (a function of wavelength) [34]:

(1) $$\int_{\Omega }\;E\left(\lambda \right)Q_{k}\left(\lambda \right)d\lambda =\rho_{k}\;.$$

Here, $\rho_{k}$ is the *k*th-channel camera response depending on $Q_{k}$. For the RGB camera, k=1,2,3, meaning three different color sensors are used, whereas for a multispectral camera, *m* sensor functions will be considered (where m>3). In this paper, we consider Ω, the range of integration, to be the visible range.

A discrete variant of Equation (1) is [34]

(2) $$Q^{T}\underline{e}=\underline{\rho }\;$$

where the columns of Q are the discretized $Q_{k}\left(\lambda \right)$’s (Q is an n×m matrix). When m=3, $\underline{\rho }={\left[\rho_{1},\rho_{2},\rho_{3}\right]}^{T}$ is an RGB vector. In the case of multispectral imaging, in this paper, we will denote the resulting camera response vector as $\underline{c}$ instead of $\underline{\rho }$ for distinction. The vectors $\underline{\rho }$ and $\underline{c}$ will always, respectively denote 3- and *m*-dimensional response vectors (where m>3).

This paper aims to study how we could improve spectral recoveries when the camera spectral sensitivities Q, are “known”. Therefore, Q is assumed to have been measured, using techniques such as a spectral-scanning monochromator [35].

### 2.2. Matrix-R

The so-called “Matrix-R” [22,31] is the matrix that projects any spectra onto the column space of Q (the projection is in the span of Q and is closest in a least-squares sense). In linear algebra, this projection matrix is written as [36]

(3) $$R=Q{\left[Q^{T}Q\right]}^{-1}Q^{T}\;.$$

Using this matrix R, we calculate the component of a given spectrum $\underline{e}$—the (actual) fundamental metamer ${\underline{e}}^{Q}$—that lies in the column space of Q:

(4) $$\begin{array}{cc} {\underline{e}}^{Q} & =R\underline{e}\\ \; & =Q{\left[Q^{T}Q\right]}^{-1}Q^{T}\underline{e}\\ \; & =Q{\left[Q^{T}Q\right]}^{-1}\underline{\rho }\;. \end{array}$$

It is clear that

1. $Q^{T}{\underline{e}}^{Q}=Q^{T}\underline{e}=\underline{\rho }$, meaning that ${\underline{e}}^{Q}$ has the same RGB sensor response as $\underline{e}$.
2. ${\underline{e}}^{Q}$ is fixed for all ${\underline{e}}^{\prime }$ (including the ground-truth $\underline{e}$) satisfying $Q^{T}{\underline{e}}^{\prime }=\underline{\rho }$, i.e., all spectra that return the same color when observed by the camera sensitivities Q.
3. ${\underline{e}}^{Q}$ can be exactly calculated given camera’s spectral sensitivities Q and the RGB sensor response $\underline{\rho }$, without the need of knowing the ground-truth $\underline{e}$.

Then, the residual, or metameric black, component of $\underline{e}$ is denoted ${\underline{e}}^{\mathrm{Null}(Q)}$ and is calculated as

(5) $${\underline{e}}^{\mathrm{Null}(Q)}=\underline{e}-{\underline{e}}^{Q}\;=\left[I-R\right]\underline{e}\;.$$

Here, I is the n×n identity matrix. The term metameric black is used because the camera’s response to this signal is zero:

(6) $$Q^{T}{\underline{e}}^{\mathrm{Null}(Q)}={\left[0,0,0\right]}^{T}\;.$$

Equivalently, in the parlance of linear algebra, we say ${\underline{e}}^{\mathrm{Null}(Q)}$ lies in the null space of the column space of Q [30] (hence the notation). And this null space is in fact the residual n−3 dimensions in the spectral space that are perpendicular to the 3-dimensional camera sensor subspace spanned by columns of Q [36,37]. The matrix [I−R] is the projection matrix for this null space.

Unlike ${\underline{e}}^{Q}$, which can be calculated directly from the RGB, ${\underline{e}}^{\mathrm{Null}(Q)}$ is unbounded by the color image formation. Indeed, ${\underline{e}}^{\mathrm{Null}(Q)}$ can be any vector in the (n−3)-dimensional null space of Q without altering the RGB observation $\underline{\rho }$.

### 2.3. Spectral Reconstruction

**Figure 3:**
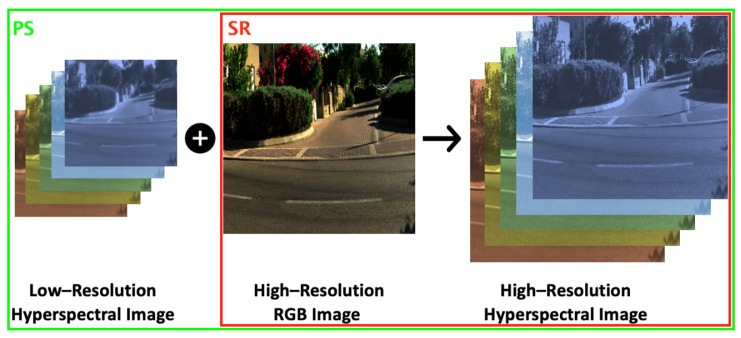
An illustration of the RGB-based hyperspectral pan-sharpening (PS; green box) and spectral reconstruction (SR; red box). The images are generated from the ICVL hyperspectral image database [40]. **Left**: The demonstration of the low-resolution hyperspectral image. **Center**: The high-resolution RGB image. **Right**: The target high-resolution hyperspectral image.

In spectral reconstruction (SR), high-resolution hyperspectral images are directly recovered from their RGB counterparts (Figure 3, red box). One of the simplest SR methods is linear regression [11], where a matrix transformation is found that maps the 3-dimensional RGBs to *n*-dimensional spectra. A simple extension to linear regression is to map each RGB to higher dimensional terms, e.g., using a polynomial expansion, [38,39]. These regression models are—in effect—a form of look-up-table since each RGB will always be mapped to the same output spectrum. Other more general one-to-one mappings reported in the literature include radial basis function regression [13], A+ sparse coding [12], and A++ sparse coding method [14]. Significantly, A++ delivers better recovery performance than many of the leading deep learning methods.

Recent SR methods are often based on machine learning and deep neural networks. Leading methods include HSCNN-D [16], HSCNN-R [16], and AWAN [17]. HSCNN-D and HSCNN-R are, respectively, the winner and runner-up of the NTIRE 2018 competition on SR [25], where the former adopted a densely-connected convolutional network [41], and the latter is based on the deep residual network architecture [42]. Then, the AWAN method is the winner of the 2020 edition of the NTIRE competition [24], where the non-local attention mechanism [43] is incorporated.

Several classical SR methods in the literature make simplifying assumptions as an aid to recovering spectra from RGBs. Beginning with Maloney and Wandell [44], early SR approaches, e.g., Ref. [45], represented spectra with a 3-dimensional linear model. With respect to this 3-D model the spectral weights—a 3-dimensional vector—were shown to be in linear relation to the recorded RGBs. It followed that spectral recovery involved simply inverting the linear relation. Morovic and Finlayson [46] developed a Bayesian framework that is based on known spectral sensitivities and complies with Matrix-R (in the sense that Matrix-R post-processing does not further improve the estimate). According to this method, for any given input RGB, all spectra with the correct fundamental metamer are shown to form a “metamer set” that acts as a constraint for Bayesian inference. The Matrix-R post-processing was applied to spectra recovered by Zhao et al. [47] to ensure the correct fundamental metamer (without considering its optimality).

The idea that any recovered spectrum should integrate to the same (or similar) RGB has recently appeared in the deep network-based SR literature. The AWAN [17] method incorporates a color (RGB) difference term in its loss function, where the RGBs of ground-truth and reconstructed spectra are calculated via Equation (2) with the camera spectral sensitivities and compared. Despite AWAN’s effort to lower the color error, it is still not completely accurate in color, as shown in [24]. This means that the fundamental metamers of the recovered spectra by AWAN still do not match with the ones derived from the RGBs. Lin and Finlayson [23] addressed the general problem of Matrix-R non-compliance in SR by restricting all algorithms to only predict the metameric black components of the ground-truth spectra while keeping the fundamental metamer components identical to the ones derived from the RGBs. Nonetheless, this approach requires retraining of the entire algorithms, and the performance improvement is not guaranteed [23].

### 2.4. Pan-Sharpening

In pan-sharpening, we wish to fuse low-resolution hyperspectral or multispectral images with a high-resolution RGB counterpart; see Figure 3. Here, left, a low-res hyperspectral image (or relatedly a multispectral image) is fused in some way with the full-resolution RGB image (middle) to, hopefully, produce a good estimated high-res image (right). We note that in the pan-sharpening literature (especially older algorithms), imaging systems often only have access to a grayscale full-res image instead of RGB. Here and throughout this paper, we only consider pan-sharpening problem guilded by RGB input.

Interestingly, many of the prior-art methods generate their full-res outputs without formally considering whether their algorithm is accurate or not. That is because these algorithms were not trained against a benchmark ground-truth dataset. Prominent examples of this approach include the Coupled Nonnegative Matrix Factorization (CNMF) [19] and the coupled spectral unmixing methods [18]. The latter method can be seen as an improvement of the former where physical constraints are placed on the spectra that are recovered.

In more recent research, e.g., Refs. [21,48], has introduced algorithms that leverage the knowledge of ground-truth data. These methods involve the use of deep neural networks (DNNs) to map RGB images combined with low-resolution spectral input to high-resolution ground-truth outputs. Once trained, these networks can be applied to unseen data for predictions. However, one significant drawback of the DNN approach is its reliance on millions of parameters, which can make the models highly complex and computationally intensive. Moreover, there is often an insufficient amount of training data available to reliably train these networks, raising concerns about generalization [14]. Another challenge arises when new training data become available or when researchers switch to different camera sensitivities [49]. In such cases, the entire network may need to be retrained, which can be a time-consuming and resource-intensive process.

There are hybrid methods such as the Model-Inspired Autoencoder (MIAE) [20], which are still based on finding the prior spectral and spatial prior to solve a Non-Negative Matrix Factorization problem on per-scene basis, while formulating the prior crafting as a deep learning problem. A key component of MIAE relevant to the research we report in this paper is that this algorithm exploits knowledge of the camera’s spectral sensitivities. As MIAE also delivers leading performance results, we will use it here as an exemplar deep learning algorithm to benchmark against.

Finally, we note that there have been methods that incorporate knowledge of the RGB camera spectral sensitivities to refine their pan-sharpening method. Most notably, Imai and Burns [50] directly applied Matrix-R compliance as a standalone pan-sharpening algorithm: the lower-resolution hyperspectral image is first resized (upsampled) to the same image dimension as the RGB image, and then, at each pixel, we replace the fundamental metamer component of the low-resolution spectrum by the one calculated from the RGB image. Essentially, our method extends the work of Imai and Burns. We also prove that post-processing with Matrix-R must always result in improved spectral estimation for any SR or PS algorithm. This is an important point as the Imai and Berns method, viewed as the vantage point of the performance afforded by today’s most effective algorithms, delivers relatively poor performance (Matrix-R alone does not suffice). Finally, we extend the Matrix-R theory so it can be more powerfully applied when we know something about the lower-dimensional spectral subspace where scene spectra lie, which is a key innovation to obtaining the best performance.

## 3. Proposed Method

### 3.1. Matrix-R Post-Processing for Improving Hyperspectral Recovery

Let us continue to use the notation $\underline{e}$ as the ground-truth spectrum at a pixel (measured by a hyperspectral imager) and $\hat{\underline{e}}$ as a spectral estimate made using a PS (pan-sharpening) or SR (spectral reconstruction) algorithm. Here, we will use the convention that the overscript ^ denotes the primary estimation from a PS or SR algorithm. Since our Matrix-R method (see Figure 2) refines an estimate to bring an estimate closer to the actual (ground-truth) spectrum, we use the double-hat ^^ to denote the refined estimate.

A priori, we can write $\underline{e}$ and $\hat{\underline{e}}$ as sums of fundamental metamers and metameric blacks (see Section 2.2):

(7) $$\left\{\begin{array}{c} \underline{e}={\underline{e}}^{Q}+{\underline{e}}^{\mathrm{Null}(Q)}\;\\ \hat{\underline{e}}={\underline{\hat{e}}}^{Q}+{\underline{\hat{e}}}^{\mathrm{Null}(Q)} \end{array}\right.\;.$$

While we do not know the ground-truth spectrum $\underline{e}$ in practice, we can still calculate ${\underline{e}}^{Q}$ from the input RGB $\underline{\rho }$, as shown in Equation (4), i.e., the *actual* unknown part of the ground-truth $\underline{e}$ is ${\underline{e}}^{\mathrm{Null}(Q)}$. Significantly, in almost all data-driven PS and SR algorithms, errors are allowed even in the fundamental metamer’s part. That is, ${\underline{\hat{e}}}^{Q}\neq {\underline{e}}^{Q}$.

In this paper, we propose that using the Matrix-R theory (summarized in Figure 2) we can get a refined estimate 1pt6.5pte_^^, which is always going to be the same or *closer* to the ground-truth $\underline{e}$ than $\hat{\underline{e}}$ (the idea of *closer* is defined in Euclidean distance, or equivalently, lower root-mean-squared error, RMSE). In mathematical terms the refinement process is written as

(8) 1pt6.5pte_^^=e_^−e^_Q+e_Q,

or equivalently,

(9) 1pt6.5pte_^^=e_Q+e^_Null(Q).

**Theorem 1.**  *The refined output, 1pt6.5pte_^^, is always as close or closer to the ground-truth $\underline{e}$ than the initial estimate $\hat{\underline{e}}$, i.e., ||e_−1pt6.5pte_^^||≤||e_−e_^|| (where ||·|| denotes the L-2 norm).*

**Proof.**  Let us denote $\hat{\Delta }=\left|\right|\underline{e}-\hat{\underline{e}}{\left|\right|}^{2}$ and 1pt5.5ptΔ^^=||e_−1pt6.5pte_^^||2. Clearly, the theorem will be proved if we prove 1pt5.5ptΔ^^≤Δ^.

First, let us consider $\hat{\Delta }$ with respect to the fundamental metamer and metameric black decomposition:

(10) $$\begin{array}{cc} \hat{\Delta } & =\left|\right|\underline{e}-\hat{\underline{e}}{\left|\right|}^{2}\;\\ \; & =\left|\right|\left({\underline{e}}^{Q}+{\underline{e}}^{\mathrm{Null}(Q)}\right)-\left({\underline{\hat{e}}}^{Q}+{\underline{\hat{e}}}^{\mathrm{Null}(Q)}\right){\left|\right|}^{2}\;\\ \; & =\left|\right|\left({\underline{e}}^{Q}-{\underline{\hat{e}}}^{Q}\right)+\left({\underline{e}}^{\mathrm{Null}(Q)}-{\underline{\hat{e}}}^{\mathrm{Null}(Q)}\right){\left|\right|}^{2}\;\\ \; & =\left|\right|{\underline{e}}^{Q}-{\underline{\hat{e}}}^{Q}{\left|\right|}^{2}+\left|\right|{\underline{e}}^{\mathrm{Null}(Q)}-{\underline{\hat{e}}}^{\mathrm{Null}(Q)}{\left|\right|}^{2}\;\\ \; & \;+2\cdot {\left[{\underline{e}}^{Q}-{\underline{\hat{e}}}^{Q}\right]}^{T}\left[{\underline{e}}^{\mathrm{Null}(Q)}-{\underline{\hat{e}}}^{\mathrm{Null}(Q)}\right]\;. \end{array}$$

Here, the cross-term is

(11) $${\left[{\underline{e}}^{Q}-{\underline{\hat{e}}}^{Q}\right]}^{T}\left[{\underline{e}}^{\mathrm{Null}(Q)}-{\underline{\hat{e}}}^{\mathrm{Null}(Q)}\right]=0\;.$$

Indeed, because both ${\underline{e}}^{Q}$ and ${\underline{\hat{e}}}^{Q}$ lie in the spectral subspace spanned by columns of Q, $\left[{\underline{e}}^{Q}-{\underline{\hat{e}}}^{Q}\right]$ is also a vector in this subspace; on the other hand, $\left[{\underline{e}}^{\mathrm{Null}(Q)}-{\underline{\hat{e}}}^{\mathrm{Null}(Q)}\right]$ is a vector lies in the null-space of Q  [36]. Substituting Equation (11) into Equation (10), we get

(12) $$\hat{\Delta }=\left|\right|{\underline{e}}^{Q}-{\underline{\hat{e}}}^{Q}{\left|\right|}^{2}+\left|\right|{\underline{e}}^{\mathrm{Null}(Q)}-{\underline{\hat{e}}}^{\mathrm{Null}(Q)}{\left|\right|}^{2}\;.$$

Next, let us examine 1pt5.5ptΔ^^:

(13) 1pt5.5ptΔ^^=||e_−1pt6.5pte_^^||2=||(e_Q+e_Null(Q))−(e_Q+e^_Null(Q))||2=||e_Null(Q)−e^_Null(Q)||2.

Following from Equations (12) and (13), it is immediate that

(14) 1pt5.5ptΔ^^=||e_Null(Q)−e^_Null(Q)||2≤||e_Q−e^_Q||2+||e_Null(Q)−e^_Null(Q)||2=Δ^.

□

Equation (14) succinctly encapsulates that the recovered spectrum post-processed using the Matrix-R method is always as close or closer to the ground-truth (compared to the original recovered spectrum returned by any PS or SR algorithm).

### 3.2. Generalization of Matrix-R Post-Processing to Multispectral Recovery

In Equations (1) and (2), we proposed to sample spectra (e.g., from 400 nm to 700 nm) at *n* wavelengths. What if Q and $\underline{e}$ were sampled at 2n or 10n wavelengths? Would that change any of the arguments? No, it would not—so long as Equation (2) remains a valid physical model of how RGBs are formed. Now, suppose we think of the columns of Q and $\underline{e}$ not as discrete spectral measurements but as some other functions of wavelength that still satisfy Equation (2). Because none of our derivations depends on the physical *meaning* of the integration/inner product step entailed in Equations (1) and (2), all the methods developed so far continue to work. We can still find fundamental metamers and metameric blacks, which lie in the space spanned by Q or in its null space, respectively. However, these metameric concepts are no longer linked to wavelength.

Let us make this abstract idea more concrete. We denote the *m*-dimensional measurements made by a multispectral imager (m>3), as $\underline{c}$. Now, we assume there is a linear relationship between $\underline{c}$ and the RGB response, $\underline{\rho }$. Our imaging model (in direct analogy to Equation (2)) is

(15) $$M^{T}\underline{c}=\underline{\rho }\;.$$

It follows that for the multispectral reconstruction and pan-sharpening problem we can still apply the same Matrix-R post-processing developed thus far. All that is changed is that the matrix R now depends on M (rather than Q). An important detail is that M is m×3 where 3<m≪n (a point we return to later).

Of course, the Matrix-R theory will apply if and only if Equation (15) is a good model of image formation. How might we find M in practice? Let C and P denote, respectively, N×m and N×3 matrices of corresponding multispectral and RGB sensor responses for *N* training stimuli. We then find M using a regularized least-squares regression:

(16) $$\underset{M}{arg min}{∥CM-P∥}_{F}^{2}+\gamma {∥M∥}_{F}^{2}\;,$$

where ${∥\cdot ∥}_{F}$ represents the Frobenius norm [51]. The user-defined γ, bounds the magnitude of M, effectively, mitigating overfitting [52,53]. Equation (16) is solved in closed form [52,54]:

(17) $$M={\left[C^{⊤}C+\gamma I\right]}^{-1}C^{⊤}P\;,$$

where I is the m×m identity matrix. The γ term is user-defined. We found that setting gamma to be a small fraction, say 0.01% of the mean variance of the data: $mean(diag\left(C^{⊤}C\right))$, works well for our purposes.

### 3.3. Matrix-R Post-Processing with a Low-Dimensional Spectral Representation

Now, let us suppose all spectra in a target hyperspectral image lies in a lower-dimensional space. We write

(18) $$\underline{e}=B\underline{b}\;,$$

where B is an n×b basis matrix (b<n), and $\underline{b}$ is a coefficient vector with *b* components. Here, for convenience for later derivations, we further assume the *orthonormalization* of the columns of B, i.e., the columns of B are normalized to unit vector and are orthogonal to each other. This can be achieved in various ways given any *b*-dimensional basis, e.g., using the Gram–Schmidt process [55]. To ease notation we will still write spectra as $\underline{e}$, where we implicitly assume the linear model assumption.

Next, the definition of fundamental metamer (and metameric black) can then be defined in terms of the interaction of the B subspace and the camera spectral sensitivities (and so we will draw attention to this fact in our notation). We point out that only the part of Q spanned by the basis B contributes to the RGB observations of any spectra written in the form of Equation (18). Indeed, since $\underline{e}$ lies in the column space of B, the part of Q perpendicular to B will have no effects in the color image formation $Q^{T}\underline{e}$ (Equation (2)). Given this prior knowledge, we can now define a new data-dependent spectral sensitivity matrix:

(19) $$\bar{Q}=BB^{T}Q\;,$$

where $BB^{T}$ is the projection matrix with respect to B (plugging B into Equation (3) returns this projector because B has orthonormal columns). We can examine the equivalence of Q and $\bar{Q}$ in color image formation by replacing Q by $\bar{Q}$ in Equation (2):

(20) $${\bar{Q}}^{T}\underline{e}=Q^{T}BB^{T}\underline{e}=Q^{T}\underline{e}=\underline{\rho }\;.$$

Here, the $BB^{T}$ projection does not alter $\underline{e}$ because $\underline{e}$ already lies in the column space of B, as shown in Equation (18). More importantly, this equivalence allows us to create a new post-processing Matrix-R method simply by following the same derivation but with $\bar{Q}$ instead of Q. That is, in analogy to Equations (3) and (4), we define a modified matrix $\bar{R}$ as

(21) $$\bar{R}=\bar{Q}{\left[{\bar{Q}}^{T}\bar{Q}\right]}^{-1}{\bar{Q}}^{T}$$

and write the actual and estimated fundamental metamer, ${\underline{e}}^{\bar{Q}}$ and ${\underline{\hat{e}}}^{\bar{Q}}$, respectively as

(22) $$\left\{\begin{array}{c} {\underline{e}}^{\bar{Q}}=\bar{R}\underline{e}=\bar{Q}{\left[{\bar{Q}}^{T}\bar{Q}\right]}^{-1}\underline{\rho }\;\\ {\underline{\hat{e}}}^{\bar{Q}}=\bar{R}\hat{\underline{e}} \end{array}\right.\;.$$

It follows that with respect to $\bar{Q}$—analogous to Equations (8) and (9)—our second Matrix-R post-processing algorithm with a low-dimensional data assumption is written as

(23) 1pt6.5pte_^^=e_^−e^_Q¯+e_Q¯=e_Q¯+e^_Null(Q¯).

**Theorem 2.**  *Assuming that spectra are in the span of an m-dimensional linear model, the refined spectral estimate 1pt6.5pte_^^, calculated using Equation (23), will always be closer to the ground-truth $\underline{e}$ than the initial estimate $\hat{\underline{e}}$, i.e., ||e_−1pt6.5pte_^^||≤||e_−e_^||.*

We do not need to formally prove the second theorem, as the original Matrix-R theorem does not limit us to use any particular spectral sensitivity matrix Q for the Matrix-R decomposition. In fact, the theorem holds for any n×3 matrix that derives RGBs from spectra, which, as Equation (20) has shown, applies to both Q and $\bar{Q}$.

#### 3.3.1. Determining the Basis B

The basis B can be calculated a priori, e.g., based on known reflectances and a known illuminant. For the PS application, we additionally have access to a low-res hyperspectral image of the scene, and we might extract a low-dimensional image from this image. A third alternative would be to calculate the basis from the high-res spectral reconstruction returned by a given algorithm. In all three cases, given a corpus of spectral measurements, it is easy to find the best least-squares optimal basis using techniques like characteristic vector analysis [44]. Importantly, there is a reasonable expectation that a low-dimensional basis will well describe spectral data. Indeed, most spectral reflectances are smooth functions of wavelength (and are often represented by six to eight basis functions). The dimensionality of observed smooth reflectances under a single non-smooth illuminant does not change.

#### 3.3.2. Extension to Multispectral Recovery

Rather than thinking about image formation in the wavelength domain, we can instead adopt Equation (15) as our image formation model (an RGB is a linear sum of the responses from a *m*-sensor imager). With respect to this imager, we can again adopt a *b*-dimensional model for spectra. According to these assumptions, we write

(24) $$\underline{c}=C\underline{b}\;.$$

Here, C is a m×b orthonormal basis matrix of responses (where, to elicit any computational advantage, we need b<m). With respect to this matrix, we can derive a new data-dependent image formation matrix:

(25) $$\bar{M}=CC^{T}M\;.$$

Then, the arguments from Section 3.2 and Section 3.3 all hold: we need only substitute, respectively, $\underline{c}$ for $\underline{e}$, $\bar{M}$ for $\bar{Q}$, and $CC^{T}$ for $BB^{T}$.

## 4. Experiments

### 4.1. Data Preparation

We will use the ICVL hyperspectral image database [40] for our experiments. ICVL consists of 201 hyperspectral images of size 1300×1392 (though a handful of images are slightly smaller) and 31 spectral dimensions (10-nanometer sampling of the visible spectrum between 400 and 700 nanometers). The original images are encoded in 12 bits, i.e., the maximal pixel value is 4095. We re-scale the encoding range to [0, 1] by dividing 4095 from the original pixel values.

In our experiments, the RGB images are generated pixel by pixel from the ground-truth hyperspectral images via Equation (2). For spectral reconstruction experiments, the CIE 1964 Color Matching Functions [56] are used as the camera spectral sensitivities to generate RGB images since this is proposed in [40], and many spectral reconstruction algorithms were developed for this definition of RGB. For our hyperspectral and multispectral pan-sharpening experiments, RGB images are generated with the camera response functions of Canon 1D Mark III, and multispectral images with Spectricity’s 16-channel multispectral camera sensitivity functions [57]. To generate the low-resolution spectral image input for PS algorithms, both hyperspectral and multispectral images are downsampled by a factor of 8 via bilinear interpolation for pan-sharpening experiments: we simulate a hyperspectral and multispectral *thumbnails* that are 1/64 the size of the original RGB image.

For both spectral reconstruction and pan-sharpening (Table 1), the root-mean-squared error (RMSE) is used as the error metric.

(26) $$RMSE=\sqrt{\frac{1}{n}\left|\right|\underline{e}-{\underline{e}}_{\mathrm{rec}}{\left|\right|}^{2}}\;,$$

where $\underline{e}$ is the ground-truth and ${\underline{e}}_{\mathrm{rec}}$ is the recovered spectra. The recovered spectrum ${\underline{e}}_{\mathrm{rec}}$ could be the primary estimate recovered by an algorithm, $\hat{\underline{e}}$, or the refined estimate found via Matrix-R post-processing, 1pt6.5pte_^^.

### 4.2. Spectral Reconstruction Results

Spectral reconstruction results are summarized in Table 2. We consider four algorithms: the leading regression SR method, A++ [14], and three leading deepnets: HSCNN-R, HSCNN-D, and AWAN [16,17]. We applied the cross-validation approach [58] and reported mean and 99 percentile recovery statistics. The mean recovery error of an image is the mean error of overall image pixels. Then, the mean error shown in Table 2 is the mean of these per image means. Similarly, the 99 percentile error is the mean of the 99 percentile recorded per image. The RMSE figures are typically small (our data is in the interval [0, 1]), so all RMSE errors in Table 2 are multiplied by $10^{3}$ for readability. In the columns in Table 2 (and all following result tables), boldface denotes the experimental condition yielding the best results.

The RMSE performance of the original algorithm (no post-processing) is shown in the top row. Applying Matrix-R post-processing yields the results recorded in the second row of Table 2. We see that the performance increment is most significant for the regression-based A++, where the mean and 99 percentile error of the original method are, respectively, 4.2 and 4.9% lower. The gains for the HSCNN networks are less but are still significant. There is a very small improvement (noticeable only in the fourth decimal place) for AWAN (which is to be expected as this network was designed to approximately recover the correct fundamental metamer).

We now adopt the linear basis assumption where, per image, the best basis of a given dimension (the “*m*”-dim) is found via a characteristic vector analysis of the original output spectral image recovered by the SR algorithms. Note this is a strong constraint as we are assuming we have access to the optimal linear basis that describes the spectra we are attempting to recover. Clearly, adopting too few basis vectors leads to an expected decrement in performance, as we see the generally ill-performed 3-dim results. However, in all cases—for all algorithms and error metrics—a linear model assumption exists that leads to better recovery performance.

For the A++ regression method, adopting Matrix-R post-processing together with a linear basis assumption results in, respectively, a 4.6 and 10.2% improvement in the mean and 99 percentile RMSE errors, which is a critical improvement that makes A++ outperform the much more complex HSCNN-R in mean performance.

### 4.3. Hyperspectral Pan-Sharpening Results

Here, we have access to the full-resolution RGB image and a 1/8-resolution hyperspectral image. We wish to fuse these two images to recover a full-resolution hyperspectral image. We consider 4 algorithms. First, we only bilinearly upsample the low-resolution hyperspectral image (this is a control for our experiments). Then we benchmark against the classical algorithms: CNMF [19] and Lanaras et al. [18]. Finally, we look at the performance of MIAE [20], one of the leading deep-net pan-sharpening algorithms. The results are summarized in Table 3.

Applying our Matrix-R post-processing, for bilinear upsampling, the mean RMSE error of 7.94 is reduced to 3.54 (more than a 50% reduction), and with a 4-dimensional linear model, the mean error was further lowered to 2.77, surpassing the performance delivered by the CNMF. Matrix-R post-processing with the best performing low-dimensional linear model also significantly improves the performance of CNMF and Lanaras (their mean RMSE errors were reduced by 29 and 14%, respectively). Benchmarked against MIAE, the performance increment is much more modest. The 99 percentile error improvements follow similar trends.

Note that here, and later in the multispectral PS section, the *m*-dim bases are found by applying characteristic vector analysis on each input low-dimensional hyper- and multispectral image.

**Figure 4:**
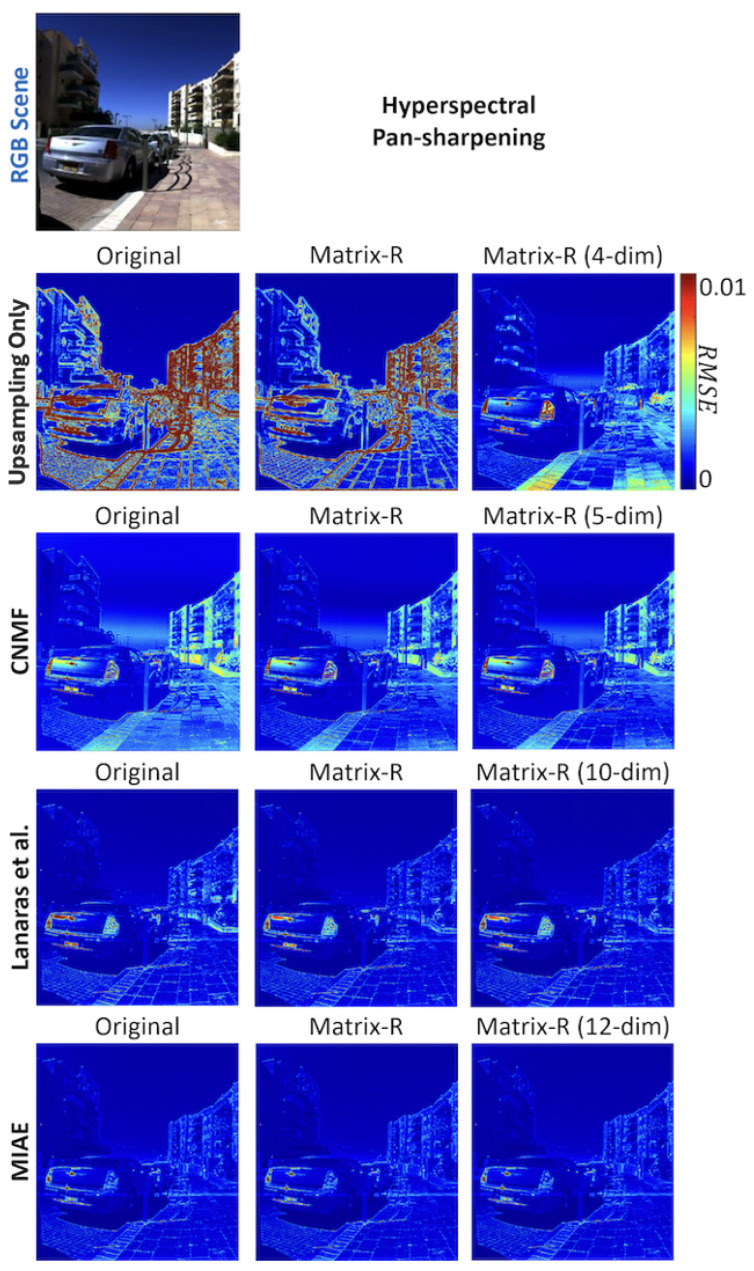
The Original, Matrix-R, and Matrix-R with a lower-dimensional spectral assumption results in RMSE error heat maps for the tested hyperspectral pan-sharpening algorithms.

In Figure 4, we visualize the recovery errors for four pan-sharpening algorithms and their post-processing by the Matrix-R algorithm and the Matrix-R with the linear basis constraint. The visualizations reflect the error statistics conveyed in Table 3 and Table 4. There is a large improvement for “upsampling only” and modest improvements for CNMF and Lanaras et al. For this image, it is hard to visually discern the improvement of post-processing for the MIAE algorithm.

### 4.4. Multispectral Pan-Sharpening Results

We conduct a proof-of-concept experiment on multispectral pan-sharpening, aiming to achieve high-resolution *m*-channel multispectral images by fusing low-resolution multispectral and corresponding high-resolution RGB images. Since this setup lacks a standard method or dataset, we perform hypothetical experiments to test Matrix-R and its lower-dimensional variants for recovering multispectral images. The RGB and multispectral images are generated by integrating the spectral images from the ICVL dataset with, respectively, the Canon 1D Mark III and Spectricity’s 16-channel multispectral camera sensitivity functions [57].

Pan-sharpening for this experimental scenario is discussed in Section 3.3 and Section 3.3.2, and we follow that methodology here. Importantly, to use this method, we need to know how RGBs and multispectral measurements are related to each other. The challenge is that there is no a priori, known direct mapping from multispectral to RGB (from $\underline{c}$ to $\underline{\rho }$ in Equation (15)), unlike in hyperspectral cases where the camera sensitivity functions directly mapping hyperspectral data to RGB are available. Thus, we must compute the transformation matrix M with regularization for multispectral pan-sharpening, i.e., Equation (17). To do that, we downscale the RGB images to match the pixels between low-resolution multispectral images for each scene. Then, we regress the multispectral images onto the RGBs and find individual matrix M per image. In solving the regression in Equation (17), there is a λ parameter controlling the penalty term. In our experiments, λ equals to 0.01% of the mean variance of the data: $mean(diag\left(C^{⊤}C\right))$.

The results are reported in Table 4, where we benchmark our multispectral pan-sharpening approach against the error found when we only bilinearly resize the multispectral image. Clearly, Matrix-R improves the upsampling-only results, and using an *m*-dimensional linear model consistently yields better or equivalent performance compared to standalone Matrix-R when m>3 (again, the m=3 case points to insufficient linear model representation of spectra as in the spectral reconstruction and hyperspectral pan-sharpening results). The best performance is achieved when m=5 for the mean, and m=4 for the 99th-percentile RMSE. In both cases, the errors are reduced by about 75%.

**Figure 5:**
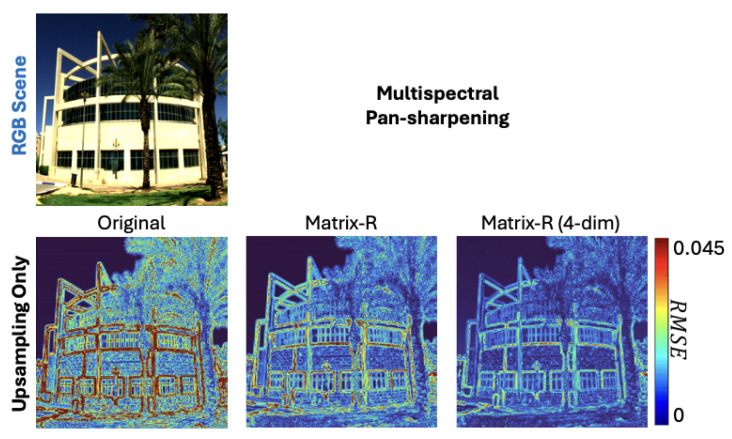
The original upsampling-only, post-processing by Matrix-R, and Matrix-R with a lower-dimensional spectral assumption results in RMSE error heat maps for multispectral pan-sharpening.

The effectiveness of our method in multispectral pan-sharpening is also illustrated in Figure 5.

## 5. Auxiliary Studies

### 5.1. Color Difference

Since the Matrix-R theorem dictates that the observed color errors result from the mismatch of fundamental metamers between ground-truth and reconstructed spectra, it is intuitive to think that the degree Matrix-R could improve the original methods should relate to the level of observed color errors. Indeed, the AWAN spectral reconstruction method [17] minimizes a color difference loss as part of its design, which also shows one of the smallest improvements when adopting Matrix-R algorithms.

**Figure 6:**
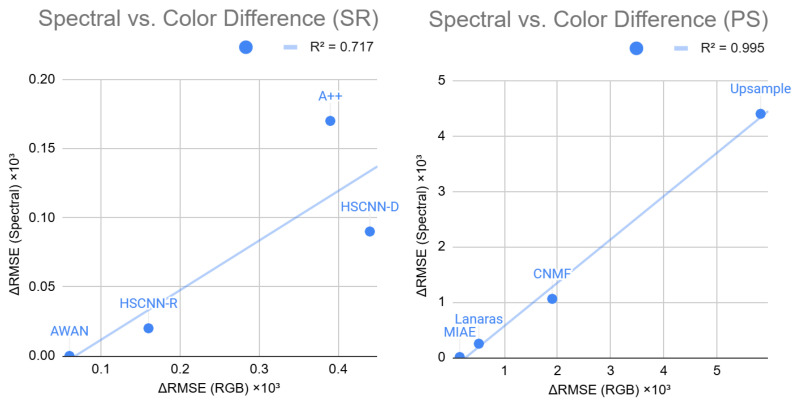
The correlation plots of spectral and color improvements (ΔRMSE (Spectral) and ΔRMSE (RGB), respectively) for spectral reconstruction (SR; **left plot**) and hyperspectral pan-sharpening (PS; **right plot**). Note that the RMSEs are scaled by $\times 10^{3}$ to be consistent with the numbers in result tables.

In Table 5 and Table 6, we show the corresponding RMSE statistics of RGB colors for the SR and PS experiments in Section 4.2 and Section 4.3. Then, Figure 6 shows the correlation scatter plots between the *spectral improvement* and *color improvement*—defined as the mean-RMSE improvements in spectral and color spaces via adopting the standalone Matrix-R algorithm and denoted as ΔRMSE (Spectral) and ΔRMSE (RGB), respectively. Evidently, we see positive correlations between color and spectral improvements, with the pan-sharpening results, shown in the right plot of Figure 6, almost aligned in a straight line ($R^{2}=0.995$). Although the spectral reconstruction (SR) result shows that it is possible that a perfect linear correlation is not ensured, the trend is still clear that methods introducing larger color errors tend to have more benefit of adopting the Matrix-R algorithm.

In Table 5 and Table 6, we also draw the attention to the “zero color errors” for all methods adopted lower-dimensional basis assumptions (*m*-dims). As we force the reconstructed spectra to lie on a particular lower-dimensional basis, we are risking that the basis might not be representative enough for the spectral data, and, subsequently, the effective spectral sensitivity matrix $\bar{Q}$ defined in Equation (19) might not be accurate in terms of color formation (Equation (20)). In contrast to this doubt, this result shows that even if the spectral basis failed to represent spectra well (in the case of m=3, as a clear example), our low-dimensional variant of Matrix-R can still maintain perfect color fidelity.

### 5.2. CAVE Dataset

With the same experimental setup, we test the efficacy of Matrix-R methods on bilinear-upsampled images from the CAVE dataset [59]. Distinct from the ICVL dataset where images are captured in the wild (indoor and outdoor), CAVE dataset includes 32 lab images. The mean and worst-case results are shown in Table 7. This result demonstrates our methods’ generalizability over different spectral datasets where images were captured under very different settings. Indeed, the CAVE dataset results show a similar trend: while standalone Matrix-R improves the spectral accuracy of bilinear upsampling, a lower-dimensional assumption can further improve the performance in both mean and worst-case performances. It is also evident that our Matrix-R method, with or without a lower-dimensional basis in place, ensures zero color errors, consistent with the results shown in Section 5.1.

### 5.3. Sensitivity Analysis to Sensor Measurement Errors

A critical component of the Matrix-R framework is the reliance on the camera’s spectral sensitivity matrix, Q, to calculate the correct fundamental metamer. In our theoretical formulation, Q is assumed to be known precisely. However, in practical applications, obtaining an exact measurement of Q is challenging. There are two primary approaches to acquiring these sensitivities: direct physical measurement and indirect estimation. The direct approach involves using a monochromator to illuminate the sensor with narrow-band light across the visible spectrum, recording the response at each wavelength interval. Conversely, the indirect approach estimates the spectral sensitivities computationally from images of calibration targets (e.g., color patches) captured under known illumination [60]. Regardless of the approach, the physical characterisation of the sensor is subject to experimental uncertainties, including calibration drift, stray light, and sensor noise. Therefore, it is useful to determine the tolerance of our post-processing method to inaccuracies in the Q matrix.

To investigate this, we conducted a sensitivity analysis by simulating realistic measurement errors. The magnitude of such errors might vary across different measurement setups and depends on numerous factors [61]. Consequently, we deliberately introduced random multiplicative noise to the ground-truth sensitivity matrix at two representative levels: ±5% (simulating a reasonable calibration error) and ±10% (representing a coarser estimation).

The results are detailed in Table 8. The first column presents the performance of the original Matrix-R post-correction using the precise camera sensitivities. The second and third columns show the results when using the ±5% and ±10% perturbed sensitivities, respectively.

The analysis reveals that the method is moderately sensitive to calibration accuracy. With a ±5% perturbation, the method still reduced the original error, although the magnitude of the improvement was approximately halved compared to the ideal case. However, at the ±10% perturbation level, the method failed to improve the mean errors and, in fact, increased them. This indicates that while the Matrix-R constraint is beneficial, it requires highly accurate sensor characterisation to be effective. This requirement is particularly stringent when the method is used as a post-processing technique following advanced AI-based algorithms (although the initial estimates of models that explicitly incorporate camera sensitivities would also naturally be degraded by such measurement errors). Since these models already produce high-quality estimates, the potential margin for improvement is narrow, making the final result highly sensitive to any inaccuracies introduced by an imperfect Q matrix.

### 5.4. Incorporating Fixed General Spectral Basis

In Section 3.3.1 we suggested that, to obtain the basis B in Equation (19) for a low-dimensional variant of Matrix-R, we conduct characteristic vector analysis on the reconstructed spectral image for SR and on the ground-truth lower-resolution input spectral image. Indeed, for each scene individually, this is an effective way to estimate the ground-truth spectral distribution. Nonetheless, how representative these spectra are depends on, respectively for SR and PS methods, the quality of the original spectral reconstruction and the actual resolution of the input low-resolution spectral images (imagine a 1/100 resolution difference instead of 1/8 in our experiments).

In this auxiliary study, we examine the effectiveness of lower-dimensional Matrix-R method with respect to two different methods of acquiring the basis B. First, we consider the Cross-Validation (CV) setting. Under CV, we randomly separate the 201 ICVL hyperspectral images into 4 groups of 50 (or 51 for one group) images. For all images in a given group, the basis is calculated via characteristic vector analysis on all pixels of all images in the other three groups. This setting mimics a real-world application process that is to train bases based on a known set of training data and apply it directly to an unseen case. We denote the basis as $B_{K=4}$, signifying we are adopting a *K*-fold cross validation with K=4.

The second setup is more general. Assuming we have a pair of reflectance and illuminant datasets that can effectively represent the reflectances and illuminations observed in general scenes, theoretically, we can derive a basis generally for all unseen data. Here, we use the SFU reflectance and illumination datasets [62] which consists of 1995 synthetic and natural reflectances and 102 usual illumination spectra. Note that some reflectances in the database were excluded due to the incomplete coverage of the concerned spectral range (400–700 nm), which left us with 1350 reflectances instead of 1995. Denoting reflectance as R(λ) and illumination spectra as L(λ), we have

(27) E(λ)=L(λ)R(λ),

The observed spectra E(λ) (or $\underline{e}$ in the discrete form, i.e., the recovery target in this paper) can be derived by wavelength-by-wavelength multiplications between illumination and reflectance spectra [34]. With this equation, we form a spectral dataset by collecting all combinations of SFU reflectances and illuminations. Then, we apply characteristic vector analysis to derive the low-dimensional basis, denoted as $B_{sfu}$.

We compare results of using $B_{K=4}$ and $B_{sfu}$ against the original per-image determination of basis B for Matrix-R refined bilinear upsampling in Table 9. Here, we see that under the cross-validation setup, while we can still find a lower-dimensional basis suggesting better mean performance compared to standalone Matrix-R, it does not happen until m=21. That is, for adopting $B_{K=4}$, we need a much higher-dimensional basis to improve from the original Matrix-R. On the other hand, using $B_{sfu}$ trained from SFU datasets did not improve the original Matrix-R’s performance. Note that since our spectral images have only 31 channels, the highest “lower” dimension is m=30, with m=31 equivalent to stand-alone Matrix-R (spectra are kept in the original dimension).

Combined, we see that under the current experimental setup, training the basis B per image performs better than using a cross-scene or general basis. The SFU result implies that $B_{sfu}$ might not be representative enough for the ICVL image dataset, and the cross-validation result likely suggests that a random selection of images used for training bases might not be a good setup in practice (grouping images based on their similarity in content and/or lighting conditions might help obtaining better-performing bases).

## 6. Discussion

Our proposed post-processing Matrix-R method can be applied in a wide context: the proposed process could be used to enhance the performance of off-the-shelves “black-box” algorithms where the algorithm source code is not available. Indeed, our theorems do not require the knowledge of the algorithm itself. We need only the input RGB and camera sensitivity information for the Matrix-R decomposition. Theorem 1 also gives the user *comfort*. It does no evil: it will *always* either improve the performance of any algorithm or, failing that, it will not reduce the algorithm’s performance.

As for Theorem 2, in our experiment, we were not given a known lower-dimensional basis that will definitely represent all spectra in each scene. This means that the interchangeability of Q and $\bar{Q}$ in Equation (20) may not hold, i.e., $\bar{Q}$ can create color error, and subsequently, error in calculating the ground-truth fundamental metamer ${\underline{e}}^{\bar{Q}}$ from the RGB input $\underline{\rho }$. Of course, we may sensibly assume that as the assumed spectral dimension *m* increases (i.e., when *m* approaching *n*, the original spectral dimension), we get more accurate low-dimensional model that represents spectra. And yet, according to our results, this cannot be the only factor that affects the optimal selection of *m*. The determination of the optimal basis dimension *m* is heavily dependent on the fidelity of the initial spectral estimate. We observed that high-performing deep learning models, such as AWAN and MIAE, typically achieve peak performance with higher-dimensional subspaces. This is likely because these models successfully recover subtle spectral nuances; applying a restrictive, low-dimensional *m* in these cases would discard valid spectral information, effectively acting as a form of over-regularisation. Conversely, simpler methods like bilinear interpolation often produce coarse spectral estimates with significant deviations from the ground-truth. For these algorithms, using a lower *m* is advantageous as it enforces a stricter prior, constraining the noisy estimates to a fundamental subspace of natural spectra and thereby filtering out gross spectral errors. Consequently, we recommend determining *m* empirically to match the specific capacity of the chosen reconstruction algorithm.

Another observation on optimal *m* is that, for some algorithms, the optimal *m* is different for mean and worst-case (99-percentile) results, and the latter generally suggests a smaller optimal *m*. This is understandable as a lower-dimensional linear model has the effect of bounding the outliers from exceeding what the underlying assumed basis can explain. Conversely, some originally more accurate pixels could be overgeneralized by the basis and lose accuracy. We can observe both effects in the “Upsampling Only” result in Figure 4. Here, in the subplot labeled “Matrix-R (4-dim)”, we see that a 4-dimensional spectral representation make the boundaries of buildings and the sky much more accurate, while loses accuracy in areas around the sidewalk.

We also observed that the magnitude of improvement from Matrix-R post-processing is inversely related to the baseline algorithm’s accuracy. Unlike “physically blind” algorithms, advanced models such as AWAN and MIAE explicitly incorporate the camera’s spectral sensitivity functions, resulting in spectral estimates with highly accurate fundamental metamers. Thus, our method offers the most significant value to algorithms that are not explicitly constrained by sensor physics.

Looking ahead, we recognize that employing real images from different cameras could present additional challenges [49,63], including image registration and varying exposure levels [24,39]. While our aim in this study was to propose a theoretical solution through simulation as a preliminary step, future research may explore these methods using actual camera setups.

## 7. Conclusions

The Matrix-R theorem teaches that, given the RGB observation and the spectral sensitivity functions of the sensors, we can certainly calculate the fundamental metamer component of the ground-truth spectrum, leaving the residual metameric black component to be uncertain. On the other hand, hyperspectral pan-sharpening algorithms seek to super-resolve low-spatial-resolution hyperspectral images given their high-spatial-resolution RGB counterparts, and spectral reconstruction (SR) algorithms recover hyperspectral images directly from the RGBs. Yet, most of these algorithms do not guarantee the exact reproduction of the fundamental metamers.

In this paper, we showed how the Matrix-R method can be used to always improve the performance of pan-sharpening and spectral recovery: we simply make sure that it has the correct fundamental metamer. And, we provide mathematical proof that this improvement will always happen. Furthermore, we developed the Matrix-R method where spectra are represented by a low-dimensional linear model.

Experiments on several historic and state-of-the-art PS and SR algorithms show that our proposed post-processing Matrix-R method always improved these algorithms. In addition, the low-dimensional linear basis variant of our theorem was shown to yield the best recovery results. Finally, our exploration of multispectral pan-sharpening reaffirms the efficacy of the Matrix-R method and its lower-dimensional variant.

## Figures and Tables

**Table 1:** List of considered SR and PS algorithms.

Spectral Reconstruction (SR)	Pan-Sharpening (PS)
1. A++ [14]	1. Imai and Burns [50]
2. HSCNN-R [16]	2. CNMF [19]
3. HSCNN-D [16]	3. Lanaras et al. [18]
4. AWAN [17]	4. MIAE [20]

**Table 2:** The RMSE ($\times 10^{3}$) spectral accuracy of the Matrix-R method (“Matrix-R”) and its lower-dimensional variants (“*m*-dim”) on **spectral reconstruction** algorithms. The best results are shown in bold font and underlined.

	A++		HSCNN-R		HSCNN-D		AWAN	
	RMSE (Spectral)		RMSE (Spectral)		RMSE (Spectral)		RMSE (Spectral)	
	Mean	99 pt	Mean	99 pt	Mean	99 pt	Mean	99 pt
Original	4.10	22.26	3.98	18.83	3.57	17.77	**2.26**	12.69
Matrix-R	3.93	21.18	3.96	18.79	**3.48**	17.64	**2.26**	**12.68**
3 dim	21.24	84.07	12.35	51.92	9.51	40.09	14.53	66.55
4 dim	4.05	**19.99**	4.35	18.82	3.78	**17.37**	3.06	14.83
5 dim	4.03	20.93	4.09	18.77	3.62	17.73	2.49	13.13
6 dim	3.93	21.01	3.97	**18.73**	3.50	17.59	2.34	12.80
7 dim	3.92	21.09	3.95	18.74	3.49	17.61	2.30	12.72
8 dim	**3.91**	21.12	**3.95**	18.75	**3.48**	17.62	2.28	12.70
⋮								
21 dim	-	-	-	-	-	-	**2.26**	**12.68**

**Table 3:** The RMSE ($\times 10^{3}$) spectral accuracy of the Matrix-R method (“Matrix-R”) and its lower-dimensional variants (“*m*-dim”) on **hyperspectral pan-sharpening** algorithms. The best results are shown in bold font and underlined.

	Bilinear Upsampling		CNMF		Lanaras et al.		MIAE	
	RMSE (Spectral)		RMSE (Spectral)		RMSE (Spectral)		RMSE (Spectral)	
	Mean	99 pt	Mean	99 pt	Mean	99 pt	Mean	99 pt
Original	7.94	62.06	3.98	14.34	1.96	10.54	1.37	6.19
Matrix-R	3.54	26.51	2.92	11.19	1.71	8.70	1.36	6.14
3 dim	13.52	63.86	13.52	63.86	13.52	63.86	13.52	63.86
4 dim	**2.77**	**11.87**	3.04	11.59	2.54	10.32	2.52	10.55
5 dim	3.33	22.37	**2.83**	**10.72**	1.90	**8.42**	1.69	7.08
6 dim	3.33	24.02	2.85	10.91	1.76	8.49	1.46	6.32
⋮								
10 dim	3.39	25.31	2.88	11.08	**1.69**	8.62	**1.35**	**6.09**

**Table 4:** The RMSE ($\times 10^{3}$) performance of the Matrix-R method as an **multispectral pan-sharpening** algorithm and its lower-dim variants (“*m*-dim”) on upsampling-only multispectral images. The best results are shown in bold font and underlined.

	Mean	99 pt
Bilinear Upsampling	7.09	52.62
Matrix-R	2.09	15.81
3 dim	32.92	167.03
4 dim	1.97	**13.93**
5 dim	**1.93**	14.54
6 dim	2.10	15.78

**Table 5:** The RMSE ($\times 10^{3}$) color accuracy of the Matrix-R method (“Matrix-R”) and its lower-dimensional variants (“*m*-dim”) on **spectral reconstruction** algorithms.

	A++		HSCNN-R		HSCNN-D		AWAN	
	RMSE (RGB)		RMSE (RGB)		RMSE (RGB)		RMSE (RGB)	
	Mean	99 pt	Mean	99 pt	Mean	99 pt	Mean	99 pt
Original	0.39	3.13	0.16	0.74	0.44	1.71	0.06	0.38
Matrix-R	0.00	0.00	0.00	0.00	0.00	0.00	0.00	0.00
All *m*-dims	0.00	0.00	0.00	0.00	0.00	0.00	0.00	0.00

**Table 6:** The RMSE ($\times 10^{3}$) color accuracy of the Matrix-R method (“Matrix-R”) and its lower-dimensional variants (“*m*-dim”) on **hyperspectral pan-sharpening** algorithms.

	Bilinear Upsampling		CNMF		Lanaras et al.		MIAE	
	RMSE (RGB)		RMSE (RGB)		RMSE (RGB)		RMSE (RGB)	
	Mean	99 pt	Mean	99 pt	Mean	99 pt	Mean	99 pt
Original	5.82	47.92	1.90	6.82	0.52	3.57	0.16	0.96
Matrix-R	0.00	0.00	0.00	0.00	0.00	0.00	0.00	0.00
All *m*-dims	0.00	0.00	0.00	0.00	0.00	0.00	0.00	0.00

**Table 7:** The RMSE ($\times 10^{3}$) performance of the Matrix-R method and its lower-dim variants (“*m*-dim”) on upsampling-only hyperspectral images from the CAVE dataset [59]. The best spectral accuracy results are shown in bold font and underlined.

	RMSE (Spectral)		RMSE (RGB)	
	Mean	99 pt	Mean	99 pt
Bilinear Upsampling	16.88	154.69	11.36	116.66
Matrix-R	8.53	73.68	0.00	0.00
3 dim	16.59	86.95	0.00	0.00
4 dim	11.61	**63.16**	0.00	0.00
5 dim	10.00	66.47	0.00	0.00
6 dim	9.20	68.84	0.00	0.00
7 dim	8.84	70.52	0.00	0.00
8 dim	8.67	71.15	0.00	0.00
9 dim	8.60	71.65	0.00	0.00
10 dim	8.54	71.94	0.00	0.00
11 dim	8.50	72.05	0.00	0.00
⋮				
16 dim	**8.43**	72.65	0.00	0.00

**Table 8:** Sensitivity analysis results for sensor measurement errors. The table reports RMSE ($\times 10^{3}$) values for the standard Matrix-R method (“Matrix-R”) and its lower-dimensional variants (“*m*-dim”) applied to **hyperspectral pan-sharpening**. Performance is compared using the original camera response functions versus versions with ±5% and ±10% random perturbations. The best results are shown in bold font and underlined.

	Original Q		5%		10%	
	Mean	99 pt	Mean	99 pt	Mean	99 pt
Bilinear Upsampling	7.94	62.06	7.94	62.06	**7.94**	62.06
Matrix-R	3.54	26.51	6.62	**11.43**	11.48	33.20
4 dim	**2.77**	**11.87**	**6.51**	16.78	15.56	36.53
5 dim	3.33	22.37	6.60	24.52	12.45	31.94
6 dim	3.33	24.02	**6.51**	25.92	11.89	**31.70**

**Table 9:** Test on using fixed spectral basis for lower-dimensional variants of our Matrix-R method (“*m*-dim”). The table reports RMSE ($\times 10^{3}$) values for the standard Matrix-R method (“Matrix-R”) and its lower-dimensional variants (“*m*-dim”) applied to bilinear upsampling images. The best results are shown in bold font and underlined.

	Per-Image B (Original)		Cross Validation $B_{K=4}$		SFU Dataset $B_{\mathrm{sfu}}$	
	Mean	99 pt	Mean	99 pt	Mean	99 pt
Bilinear Upsampling	7.94	62.06	7.94	62.06	7.94	62.06
Matrix-R	3.54	26.51	3.54	26.51	**3.54**	**26.51**
3 dim	13.52	63.86	8.45	23.37	14.88	43.68
4 dim	**2.77**	**11.87**	4.84	**18.43**	12.24	36.25
5 dim	3.33	22.37	4.65	26.11	12.16	36.03
6 dim	3.33	24.02	4.13	26.01	8.23	29.38
7 dim	3.37	24.73	3.92	25.89	7.96	29.12
8 dim	3.38	25.07	3.80	25.99	7.06	27.76
⋮						
21 dim	3.48	26.06	**3.49**	26.11	3.86	26.48
⋮						
30 dim	3.54	26.48	3.54	26.51	3.58	26.54

## Data Availability

Two publicly available datasets were used in this study. First, BGU ICVL Hyperspectral Dataset, which can be accessed via: https://icvl.cs.bgu.ac.il/pages/researches/hyperspectral-imaging.html (accessed: 22 July 2025). Second, the CAVE Multispectral Image Dataset, which can be accessed via: https://cave.cs.columbia.edu/repository/Multispectral (accessed: 1 December 2025).
